# Evaluating Anticancer and Immunomodulatory Effects of *Spirulina* (Arthrospira) *platensis* and Gamma-Tocotrienol Supplementation in a Syngeneic Mouse Model of Breast Cancer

**DOI:** 10.3390/nu13072320

**Published:** 2021-07-06

**Authors:** Hemavathy Subramaiam, Wan-Loy Chu, Ammu Kutty Radhakrishnan, Srikumar Chakravarthi, Kanga Rani Selvaduray, Yih-Yih Kok

**Affiliations:** 1 School of Medicine, International Medical University, Kuala Lumpur 57000, Malaysia; 2 School of Postgraduate Studies, International Medical University, Kuala Lumpur 57000, Malaysia; wanloy_chu@imu.edu.my; 3 Jeffrey Cheah School of Medicine and Health Sciences, Monash University Malaysia, Bandar Sunway 47500, Malaysia; Ammu.Radhakrishnan@monash.edu; 4 Faculty of Medicine, Bioscience and Nursing, MAHSA University, Bandar Saujana Putra 42610, Malaysia; Srikumar@mahsa.edu.my; 5 Product Development and Advisory Services Division, Malaysian Palm Oil Board, Bandar Baru Bangi 43000, Malaysia; krani@mpob.gov.my; 6 School of Health Sciences, International Medical University, Kuala Lumpur 57000, Malaysia; yihyih_kok@imu.edu.my

**Keywords:** breast cancer, *Spirulina*, tocotrienol, immunomodulatory, synergistic, metastasis

## Abstract

Nutrition can modulate host immune responses as well as promote anticancer effects. In this study, two nutritional supplements, namely gamma-tocotrienol (γT3) and *Spirulina,* were evaluated for their immune-enhancing and anticancer effects in a syngeneic mouse model of breast cancer (BC). Five-week-old female BALB/c mice were fed *Spirulina*, γT3, or a combination of *Spirulina* and γT3 (*Spirulina* + γT3) for 56 days. The mice were inoculated with 4T1 cells into their mammary fat pad on day 28 to induce BC. The animals were culled on day 56 for various analyses. A significant reduction (*p* < 0.05) in tumor volume was only observed on day 37 and 49 in animals fed with the combination of γT3 + *Spirulina*. There was a marked increase (*p* < 0.05) of CD4/CD127^+^ T-cells and decrease (*p* < 0.05) of T-regulatory cells in peripheral blood from mice fed with either γT3 or *Spirulina.* The breast tissue of the combined group showed abundant areas of necrosis, but did not prevent metastasis to the liver. Although there was a significant increase (*p* < 0.05) of MIG-6 and Cadherin 13 expression in tumors from γT3-fed animals, there were no significant (*p* > 0.05) differences in the expression of MIG-6, Cadherin 13, BIRC5, and Serpine1 upon combined feeding. This showed that combined γT3 + *Spirulina* treatment did not show any synergistic anticancer effects in this study model.

## 1. Introduction

Breast cancer (BC) is the most common cancer among women. It is estimated that 627,000 women worldwide died from this disease in 2018 alone [1]. Breast carcinoma can be stratified into different entities based on clinical behavior, histological features, and biological properties [2]. Some of the risk factors associated with BC include genetics [3], obesity [4], hormone replacement therapy [4], and having no children or having them after the age of 30 [5]. Symptoms of BC include swelling, redness or other visible differences in one or both breasts such as an increase in size or change in the shape of the breast, presence of lumps, and nipple discharge other than breast milk [6]. The BC can metastasize to distant organs such as bone [7], the lungs [8], liver [9], and brain [10]. In fact, most BC deaths are not due to the primary tumor itself, but are the result of metastasis [11]. Metastasis of cancer results from a sequential molecular cascade through which the cancer cells spread from the primary tumor site to distant anatomical sites, where they proliferate and create secondary neoplastic foci [12]. The cadherin family plays a crucial role in mediating cell-to-cell adhesion, and also exert a dominant role in metastasis of BC [13].

The initiation and progression of tumor cells elicit strong inflammatory responses. Inflammation and immunity are inherent characteristics of cancer [14]. According to the current literature, immune cells that are crucial in the fight against cancers include T-helper-1 (Th1) cells, which are CD4^+^ T-cells that produce IFN-γ, CD8^+^ cytotoxic T-lymphocytes (CTL), mature dendritic cells (DC), NK cells, and macrophages [15,16,17]. These cells can generate anti-tumor responses, which are useful in eliminating tumors. In contrast, CD4^+^ Th2 cells and CD4^+^ T-regulatory (Treg) cells can promote tolerance to tumors and induce immunosuppression, supporting tumor growth and progression [15,16,17,18]. Treatment options for BC includes surgery [19], radiation therapy [20], endocrine therapy [20], and chemotherapy [21]. However, many of these treatment options are associated with side-effects, such as decreased sensation in breast tissue, soreness, itching, peeling or redness in the treated area [22], hair loss, gastrointestinal disturbances, depressed immunity, and neutropenia [23]. As such, treatments with no or lesser side-effects or cytotoxic effects would be more beneficial for cancer patients. One such therapy that is rapidly gaining recognition as a potential treatment option for cancer is combination therapies that also use plant-based chemical compounds known as nutraceuticals, which have lower side-effects and toxicity. Amongst these nutraceuticals, tocotrienols (T3) were reported to possess strong anticancer effects. Tocotrienols are unsaturated vitamin E analogues found in several natural sources, such as palm oil, rice bran, and annatto seeds [24]. There are two major types of vitamin E, i.e., tocopherol (Toc) and T3, which exist naturally in four isoforms, which are alpha (α), beta (β), delta (δ), or gamma (γ). T3 can suppress the proliferation of cancer cells as well as induce apoptosis [25]. Furthermore, various studies have shown that T3 exerts anticancer effects by causing cell cycle arrest through inducing the expression of cell cycle inhibitory proteins and decreasing some cyclin-dependent kinases (CDK) [25,26,27]. T3 also induces apoptosis through the TGFβ-Fas-JNK-signaling pathways in human fibroblast T-cells [28]. Cyclin-dependent kinases (e.g., CDK2, CDK4, and CDK6) and their inhibitors, such as p21, p27, and p53 [26,29], also inhibit the proliferation of cancers cells. Another nutraceutical that is readily available on the market, and reported to have less cytotoxic effects following human consumption, is *Spirulina,* a microscopic and filamentous cyanobacterium (blue-green alga) [30]. *Spirulina* was reported to have anticancer, anti-inflammatory, antioxidant, and immunomodulatory properties [30]. The anticancer effects of *Spirulina* were reported to induce mitochondrial dysfunction through the up-regulation of Bax (Bcl2-associated X-protein) and BAD (Bcl-2 related family member). The latter promotes cell death, and its function is regulated by phosphorylation [31]. As for immune protection, *Spirulina* was shown to increase the proliferation of spleen cells without affecting thymic-derived T-cells [32]. *Spirulina* was shown to enhance the production of IL-1 by murine peritoneal macrophages [32], increase phagocytic activity of macrophages in chickens [33], and increase the production of IFN-γ by human NK cells [34]. Combination therapy with nutraceuticals could be beneficial as nutraceuticals generally have no or minimal side-effects. Combined *Spirulina* and tocotrienol (T3) supplementation may be used as a potential treatment option for BC as both nutraceuticals exhibit anticancer and immunomodulatory properties. To date, there are no reports evaluating the anticancer and immunomodulatory effects of combinatory treatment using both *Spirulina* and T3 supplementation. This study was undertaken to investigate the anticancer and immunomodulatory effects of combined *Spirulina* and T3 against BC using a BC syngeneic mouse model.

## 2. Materials and Methods

### 2.1. Spirulina

Food grade *Spirulina* (*Arthrospira*) *platensis* (hereafter referred to as *Spirulina*) powder was obtained as a gift from Earthrise Natural, USA. The major constituents of the *Spirulina* powder, as stated in the product sheet, include total carotenoids (≥ 370 (mg%)), β-carotene (≥120 (mg%)), phycocyanin (≥10%), crude protein (≥55%), and chlorophyll (≥0.9%).

### 2.2. Tocotrienol

The gamma-tocotrienol (γT3) (97%, oil form) was obtained as a gift from Davos Pharmaceuticals Pte Ltd., Singapore.

### 2.3. Breast Cancer Cell Line

The 4T1 mouse mammary cancer cell line was purchased from the American Type Culture Collection (ATCC) (ATCC, Rockville, MD, USA). The cells were cultured in the medium recommended by the ATCC (RPMI 1640 supplemented with 10% fetal bovine serum (FBS) (GIBCO/Invitrogen)).

### 2.4. Animals

Five-week-old female BALB/c mice were purchased from a local supplier (Chenneur Suppliers, Kuala Lumpur, Malaysia). The animals were housed in the animal holding facility (AHF) at the International Medical University (IMU, Kuala Lumpur, Malaysia). The animals were allowed to acclimatize for 7 days before experimental procedures began. All animals appeared healthy and exhibited normal eating patterns before the experimental procedures. All experiments with animals were performed in accordance with the international animal use guidelines approved by the Joint Committee on Research and Ethics, IMU (IMU 258/2012).

### 2.5. Experimental Design

After the acclimatization period, the mice were randomly assigned into four groups. The mice were fed daily through oral gavage with 50 µL of soy-oil (i) vehicle (control); (ii) 50 mg/kg body weight of *Spirulina*; (iii) 1 mg/day of γT3; or (iv) 50 mg/kg body weight of *Spirulina* and 1 mg/day of γT3 for 56 days (Table 1). On day 28, all the animals received a single injection of 100 µL of 4T1 cells (1 × 10^5^ cells/mL) into their mammary fat pad to induce BC [35]. The animals were culled on day 56, and various tissues were taken for analyses.

### 2.6. Body Weight and Tumor Volume

Animal weight and tumor size were recorded every three days until the animals were sacrificed. Tumor volume was calculated using a formula that was previously described (V = 0.52 × width × length; V refers to tumor volume (mm^3^), width and length refers to the short and long diameter of tumor (mm)) [36]. At autopsy, the tumor, lungs, liver, kidneys, and heart were collected and preserved in 10% formalin solution for histopathology studies. A portion of the tumor was snap-frozen and stored at −80 °C for gene expression studies.

### 2.7. Processing and Storage of Blood Samples

A cardiac puncture was performed on the sacrificed mice to withdraw peripheral blood. The blood was collected into a heparin-tube for immunophenotyping analysis. Plasma was isolated using centrifugation (2795× *g* for 10 min at 4 °C) for various biochemical analyses.

### 2.8. Immunophenotyping

The expression of various cell surface markers (CD4, CD8, CD25, CD127, and CD73) on the peripheral blood leukocytes were analyzed using flow cytometry. Briefly, 500 µL of blood collected via cardiac puncture was transferred into appropriately labelled tubes. Then, 2 mL of red blood cell lysis solution (eBioscience, San Diego, CA, USA) was added to each tube. The tubes were gently vortexed and incubated in the dark at room temperature for 3–10 min. The lysis activity was stopped by adding 1× cold phosphate-buffered saline (PBS). The cells were recovered by centrifugation (350× *g* for 5 min). The supernatant was aspirated and discarded. The remaining pellet was re-suspended in 200 µL of sheath fluid (eBioscience, San Diego, CA, USA). Then, 1.0 µL of appropriate fluorochrome-conjugated monoclonal antibody was added to the sample, and the tube was incubated in the dark at room temperature for 20–40 min. Following this, the samples were washed with 300 µL of wash buffer (PBS), and the cells were recovered by centrifugation (350× *g* for 5 min). The supernatant was discarded, 500 µL of sheath fluid was added to each tube, and the sample was analyzed using a flow cytometer (FACS Calibur, Becton-Dickinson, Franklin Lakes, NJ, USA). Data from 10,000 cells were acquired from each sample for analysis. The data collected was analyzed using Cell-Quest software. Dot-plots for the respective fluorochromes were obtained from the gated population for each sample.

### 2.9. Histopathological Analysis

The primary tumor, the lungs, liver, heart, and kidneys, stored in 10% formalin, were processed for histopathological studies. Briefly, the organs were transferred into appropriately labelled small cassettes. The cassettes were placed in an automatic tissue processer (Leica TP1020 automatic tissue processor, Leica, Wetzlar, Germany) and processed for histopathological studies. The paraffin-impregnated tissues were embedded into paraffin embedding media and cast into tissue blocks using a tissue embedding machine (Leica tissue embedding machine, Wetzlar, Germany). The tissue sections were deparaffinized by placing the slides in xylene solution and rehydrated through descending ethanol concentrations to water before staining with hematoxylin and eosin (H&E) stains. After removing excess stains, the tissue sections were dehydrated via ascending concentrations of ethanol and cleared in xylene substitute-X solution. The slides were then mounted with distyrene plasticizer xylene (DPX) and covered with coverslips. The slides were examined using a bright-field microscope (Nikon Eclipse 80ί (CF160), Kanagawa, Japan), with a 12.0-megapixel resolution camera, at various magnifications (100×, 200× and 400×) and the relevant images were captured.

### 2.10. Gene Expression Analysis

For RNA extraction from the tumor tissue, 1 mL of Tripure isolation reagent (Roche Diagnostics, Mannheim, Germany) was added to the frozen tumor tissue (50 mg), and the tube was mixed thoroughly. The sample was centrifuged, and the quality of the RNA obtained was evaluated based on an optical density (OD) ratio of 260 nm: 280 nm, which should be between 1.8 and 2 [37], as well as an OD ratio of 260 nm:230 nm, which should be ≥1.8 [37] for real-time PCR amplification. The RNA concentration was determined by measuring absorbance at 260 nm, 280 nm, and 230 nm using a NanoQuant plate (Tecan). The target gene sequences for murine mitogen-inducible gene 6 (MIG-6), Cadherin 13 (Cdh13), Baculoviral IAP repeat containing 5 (BIRC5), serpin family E member 1 (Serpine1), as well as the reference genes, phosphoglycerate kinase-1 (Pgk1) and proteasome subunit beta type-2 (Psmb2), were downloaded from the gene bank to facilitate the designing of primers to carry out the two-step reverse-transcription quantitative PCR (RT-qPCR). A commercial primer design software (PREMIER Biosoft International, Palo Alto, CA, USA) was used to screen potential primer sets to ensure that they had similar annealing temperatures (≤60 °C) and did not produce dimers and hairpins. Primer pairs were also tested for specificity using the BLASTN (NCBI) program. All the primers were synthesized commercially (IDT Integrated DNA Technologies, Singapore). The melting curve analysis for (A) BIRC5, (B) Cadherin 13, (C) MIG6, (D) Serpine1, (E) pgk1, and (F) psmb2 are provided as Figure S1. Quantitative PCR was performed to compare mRNA expression levels from tumor tissues from the non-treated (control) versus the three treated groups (*Spirulina* alone, γT3 alone, or combined Spirulina + γT3). The RNA from all samples were amplified using two-step RT-qPCR using a thermocycler (LightCycler^®^ 480 Real-Time Detection System, Roche Diagnostics). The qPCR PCR was performed using the LightCycler 480 SYBR Green I Mastermix (Roche Diagnostics, Mannheim, Germany).

### 2.11. Statistical Analysis

All data were analyzed using SPSS Statistics version 20. All the values were presented as the mean ± SEM of the six mice per group. The results were analyzed using the analysis of variance (ANOVA) statistical test followed by Tukey’s post-hoc test in the SPSS (version 20). A *p*-value of less than 0.5 (*p* < 0.05) was considered significant (95% confidence interval) compared to the control group.

## 3. Results

### 3.1. Body Weight and Tumor Volume

**Figure 1:**
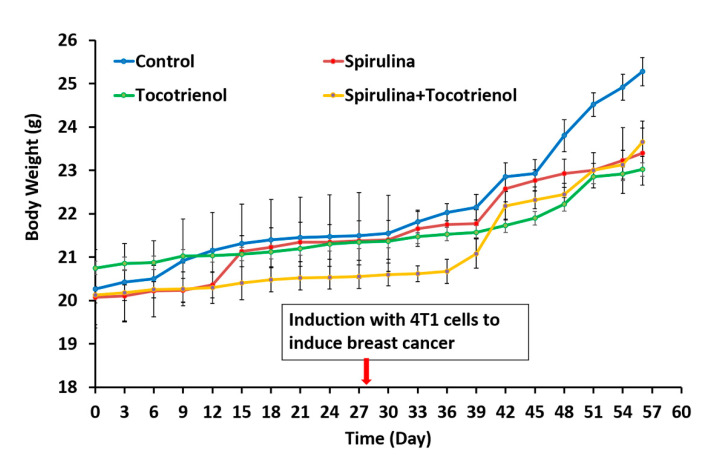
Changes in body weight before and after tumor induction in mice fed *Spirulina,* tocotrienol, and a combination of tocotrienol and *Spirulina*. Body weight of each mouse was recorded every three days from day 0 to day 56. Data expressed as mean ± standard error min (SEM) of six mice per group (*n* = 6). There were no significant differences in the body weight of animals from the control and treatment groups. Control: vehicle (soy oil); *Spirulina* (50 mg/kg body weight); γT3 (1 mg/day); and combination of γT3 (1 mg/day) and *Spirulina* (50 mg/kg body weight).

**Figure 2:**
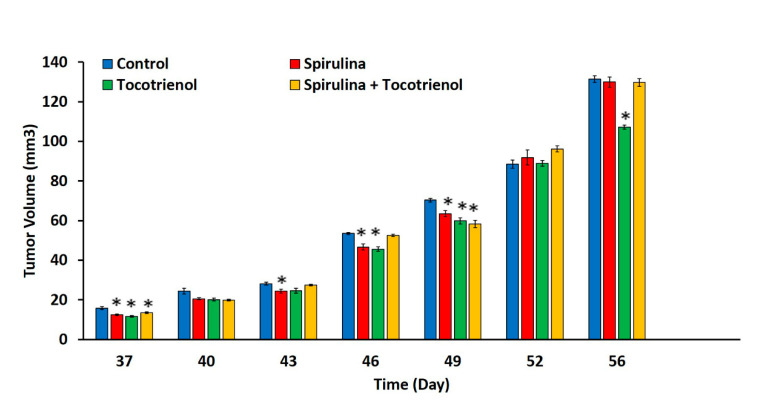
Tumor volume in mice fed *Spirulina*, γT3, and a combination of *Spirulina* and γT3. Diameter of tumor volume was measured every three days from day 37 to day 56. Data expressed as mean ± standard error mean (SEM) of six mice per group (*n* = 6). * *p* < 0.05: control versus treated group. Control: vehicle (soy oil); *Spirulina* (50 mg/kg body weight); γT3 (1 mg/day); and combination of γT3 (1 mg/day) and *Spirulina* (50 mg/kg body weight).

There were no differences (*p* > 0.05) in the body weight at day 56 amongst all the study groups (*Spirulina* (23.4 ± 0.74 g); γT3 (23.02 ± 0.74 g); and combined treatment (23.65 ± 0.33 g)) compared to the control group (25.28 ± 0.33 g) (Figure 1). The animals were induced with BC at day 28 and a tumor was palpable 9 days’ post-inoculation. The effects of supplementation of *Spirulina*, γT3 or combined treatment appeared to be quite varied. For instance, there were marked differences in tumor volume (*p* < 0.05) observed in the treated mice on day 37 (*Spirulina* (12.49 ± 0.51 mm^3^); γT3 (11.67 ± 0.33 mm^3^); and combined treatment (13.5 ± 0.38 mm^3^) and day 49 (*Spirulina* (63.5 ± 1.46 mm^3^); γT3 (59.86 ± 1.6 mm^3^); and combined treatment (58.31 ± 1.91 mm^3^) when compared to the control group (day 37: 15.83 ± 0.75 mm^3^; and day 49: 70.35 ± 0.9 mm^3^) (Figure 2). However, on day 43, a significant reduction in tumor volume was only observed in mice fed with *Spirulina* alone (24.4 ± 1 mm^3^) compared to the control group (28.17 ± 0.72 mm^3^). In contrast, on day 46, supplementation with *Spirulina* or γT3 alone significantly reduced tumor volumes (*p* < 0.05) compared to the control (Figure 2). On day 56, only γT3 supplementation showed a significant reduction (*p* < 0.05) in tumor volume compared to the control group (Figure 2).

### 3.2. Immunophenotypic Expression

**Figure 3:**
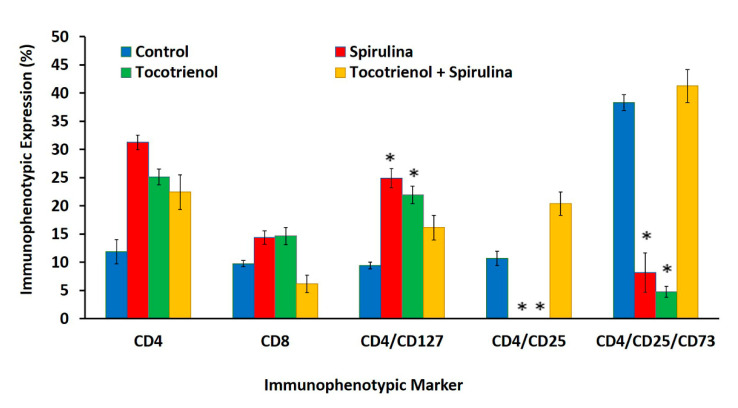
Flow cytometry analysis showing levels of Th cells (CD4^+^), CTL (CD8^+^), Th cells that secrete IL-7 (CD4^+^/CD127^+^), Treg cells (CD4^+^/CD25^+^), and Treg cells that expressed CD73 enzyme (CD4^+^/ CD25^+^/CD73). Mice in the control group were fed the vehicle for 56 days while mice in the experimental group were supplemented with tocotrienol and *Spirulina*. Data expressed as mean ± standard error mean (SEM). (*n* = 6 for control, *Spirulina*, γT3 + *Spirulina*, and *n* = 5 for γT3) (* *p* < 0.05 compared to the control group).

There were no differences (*p* > 0.05) in the percentage of CD4^+^ or CD8^+^ T-cell populations amongst all the treated groups (*Spirulina*, γT3, or combined treatment) compared to the control (Figure 3). However, the percentage of Th cells that secrete IL-7 (CD4^+^/CD127^+^) were higher (*p* < 0.05) in mice fed with just γT3 (21.9 ± 1.55%) or *Spirulina* (24.9 ± 1.69%) compared to mice from the control group (9.45 ± 0.61%). There was a ten-fold reduction in the percentage of Treg cells (CD4^+^/CD25^+^) in mice that were fed with either γT3 or *Spirulina* alone when compared to the control or combined treatment groups (Figure 3). There was also a marked reduction (*p* < 0.05) of a subset of Treg population, which expresses the CD73 enzyme (CD4^+^/CD25^+^/CD73) in mice fed with either γT3 or *Spirulina*, but there was a marked increase of this Treg subset in mice from the control or combined treatment groups (Figure 3). Representation of dot plot distribution attached as Figure S2.

### 3.3. Histopathology

**Figure 4:**
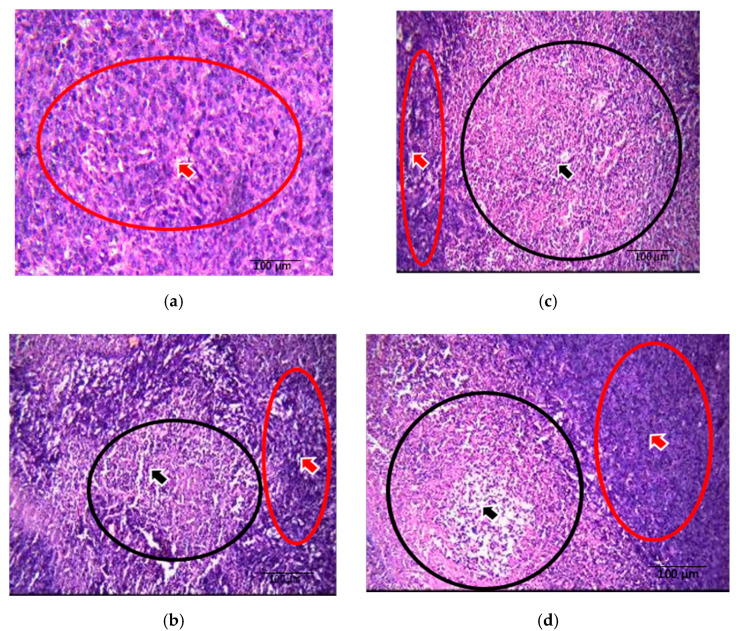
Comparison of tumor necrosis in breast tissue upon tocotrienol and *Spirulina* treatment. Photomicrographs (H&E, 200×) of breast tumor tissue from mice from the different treatment groups. The (**a**) control (non-treated), (**b**) *Spirulina* (50 mg/kg body weight), (**c**) γT3 (1 mg/day), and (**d**) combination of γT3 and *Spirulina* treated mice. Red colored arrow and circle indicate viable tumor cells, while the black colored arrow and circle indicate necrotic tumor cells.

**Figure 5:**
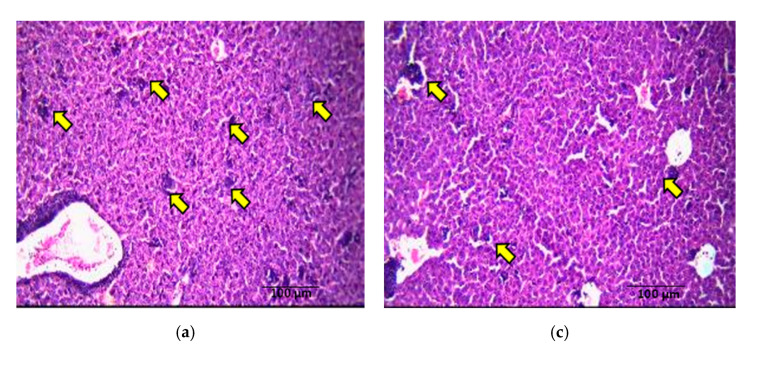

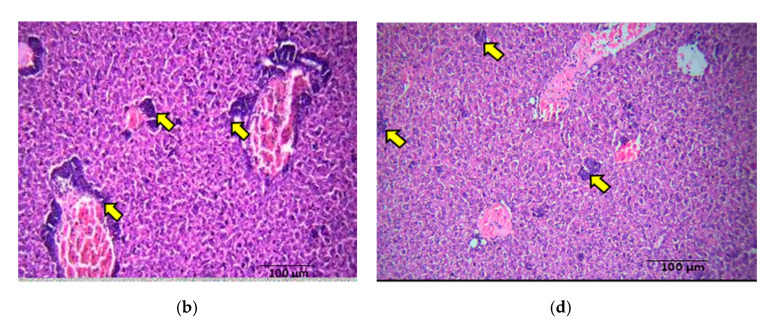
Comparison of metastasis from primary tumor site to the liver tissue upon γT3 and *Spirulina* treatment. Photomicrograph (H&E, 200×) comparing liver tissue (**a**–**d**) between (**a**) control (non-treated), (**b**) *Spirulina* (50 mg/kg body weight), (**c**) γT3 (1 mg/day), and (**d**) combination of γT3 and *Spirulina* treated mice. Arrow indicates metastasized tumor cells.

There were large and pleomorphic cells with hyperchromatic nuclei and dense cytoplasm arranged in clusters and sheets in tumor sections from control mice. These sections also showed a large primary breast adenocarcinoma (shown by arrow), which was poorly differentiated (Figure 4a). Tumor sections from the *Spirulina-*fed group displayed poorly differentiated adenocarcinoma with abundant necrosis areas (shown by arrow) in the central areas (Figure 4b). In the γT3 treated group, the breast tissues showed predominantly tumor necrosis (shown by arrow) with sheets and islands of homogenous eosinophilic necrotic cells and little proliferating tumor tissue (Figure 4c). The breast tissue from mice fed a combination of γT3 and *Spirulina* showed abundant necrosis areas (shown by arrow) (Figure 4d). Clusters of tumor cells could be seen around the central vein and in the parenchyma (shown by arrow) in the liver section from animals in the control group (Figure 5a). Metastasis to the liver was also seen in sections from *Spirulina*-fed mice (Figure 5b). Liver sections from γT3-fed mice showed metastatic deposits around the blood vessels and in the parenchyma (Figure 5c). A similar finding was observed in the liver of mice fed a combination of γT3 and *Spirulina* (Figure 5d). There was no metastasis seen in the heart, lung, or kidney sections from mice in any of the study groups.

### 3.4. Gene Expression

**Figure 6:**
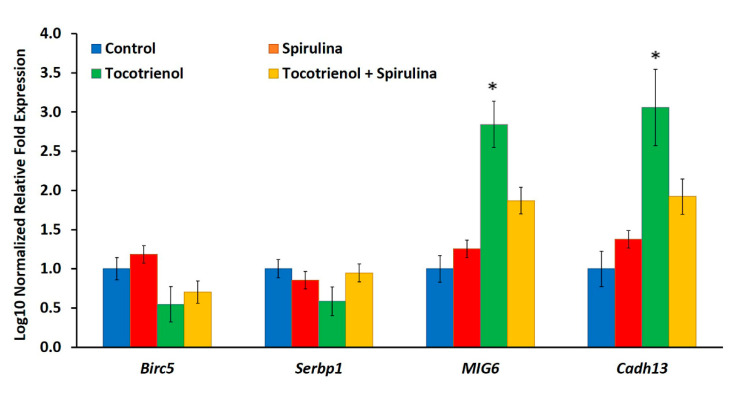
Comparison of BIRC5, Serpine1, MIG6 and Cadherin13 gene expression levels between the control (untreated), *Spirulina,* γT3, and combined *Spirulina* and γT3 treatment groups based on quantitative PCR analysis. Error bars represent standard error (SE) value, *n* = 3. (* *p* < 0.05 compared to control).

The expression of the MIG6 (>two-fold) and Cadherin 13 (three-fold) genes were up-regulated (*p* < 0.05) in tumor tissue from γT3-fed mice (Figure 6). In contrast, there was no change (*p* > 0.05) in the expression of BIRC5 and Serpine-1 genes in tumor tissues from all treated groups when compared to the control group (Figure 6).

## 4. Discussion

There was no significant difference in mouse body weight from treatment groups compared to the control group. The tumor was palpable one week after tumor induction (day 37) in mice from all four groups. The tumor volume was significantly lower in animals fed daily with γT3, *Spirulina*, or a combination treatment in the early stages compared to the control. This might be because of the effectiveness of γT3, *Spirulina*, or combination treatment in suppressing tumor growth in the early stage of cancer development, especially as the mice were fed with the test compounds 28 days before tumor induction. It is also possible that the supplements may have helped enhance the animals’ immune system to help them eliminate the tumor cells, which can result in reduced tumor mass. Similar observations have been reported previously with *Spirulina* [32,33,38] and T3 [39,40,41], possibly due to their immunomodulatory activities. On days 40, 43, 46, and 49, there was a reduction in tumor volume in all the treated groups with a significant reduction in mice from the *Spirulina-*treated group on days 43, 46, and 49. Mice from the γT3-fed group showed a significant reduction in tumor volumes on days 37, 46, and 56. The reduction in tumor volume indicates that the test nutraceutical could inhibit tumor growth. Among the three treatments, γT3 showed the most consistent reduction in tumor growth in comparison to the control group.

Cell-mediated immunity is an important adaptive immune response to combat tumors in the cancer microenvironment [42], which includes the actions of Th cells (CD4^+^), CTL (CD8^+^), and Treg cells (CD4^+^/CD25^+^). The CD8^+^ T-cell and CD4^+^ T-cell are the principal weapons of immunity against cancer [43], but prolonged immune responses can result in extended tissue damage, resulting in fatal immunopathology [44]. The balance between pro- and anti-inflammatory states of immune response could be regulated by Treg cells, which serve as gate-keepers of the immune system. Treg cells often accumulate in the tumor microenvironment [45]. Increased Treg cells in the tumor microenvironment can suppress anti-tumor T-cell responses, which can be associated with tumor progression [46,47,48]. Immunophenotyping showed that Th cells expressing the IL-7 receptor (CD4^+^/CD127^+^) were significantly higher in peripheral blood from *Spirulina*-fed mice. These mice also showed a significant reduction of Treg cells (CD4^+^/CD25^+^) and Treg cells that express CD73 enzyme compared to the control group. These findings indicate that *Spirulina* supplementation may have induced immune protective effects against BC as there was an increase in IL-7 secreting Th cells, promoting the production of pro-inflammatory and lymphocyte growth cytokines to enhance immune activity [49,50]. In addition, the Treg populations were found to be reduced, which could have beneficial effects on the host immune system. The results indicate that *Spirulina* may modulate the T-cell populations and enhance anticancer effects in this mouse model. A study using a mouse BC model reported that phycocyanin from *Spirulina* promoted proliferation of thymocytes and splenocytes from BC-induced mice compared to the control group [51]. These findings also suggest that *Spirulina* had immune-enhancing effects by increasing the populations of immune cells involved in combatting cancer. A similar enhancement of immune response was also observed in mice fed with γT3 in the present study. There was a significant increase in Th cells (CD4^+^/CD127^+^) as well as a significant decrease in Treg cells (CD4^+^/CD25^+^ and CD4^+^/CD25^+^CD73^+^) compared to the control group. These results suggested that, in this study model, γT3 enhanced immune-protection mediated by T-cells against BC. Such results are in accordance with other reports on the immunomodulatory effects of T3. For instance, in a study using athymic mice with BC, it was reported that supplementation with tocotrienol-rich fraction (TRF) up-regulated the expression of CD74/li and CD59 genes and down-regulated the expression of the IgG Fc receptor gene [39]. The CD4/li gene was reported to play a central role in the biological functions of major histocompatibility complex (MHC) class 1 proteins [52], while the IFITM-1 gene was shown to be involved in transduction of anti-proliferative and adhesion signals [53]. The binding of CD59 host cell membrane inhibited the action of the membrane attack complex (MAC) formed following activation of the complement system [54], which protects host cells [39]. These findings indicate that T3 might enhance anti-tumor effects by improving host immune responses. Combination treatment with γT3 and *Spirulina* did not show any significant difference in the T-cell populations. This may be due to a lack of synergy between γT3 and *Spirulina*. Furthermore, no previous studies reported on immunomodulatory effects when γT3 and *Spirulina* were used in combination.

Metastasis results from the dissemination of tumor cells from the primary neoplasm to a distant organ and the subsequent adaptation to a foreign tissue microenvironment [12,55]. The development of metastasis disease often signals poor prognosis, giving rise to increased morbidity and mortality [12]. In this study, tumor sections from the control group were large and pleomorphic with hyperchromatic nuclei and dense cytoplasm arranged in clusters and sheets, while tumor sections from *Spirulina-*treated groups showed primarily poorly differentiated adenocarcinoma with some areas of necrosis, indicating that there could be killing off of some of these tumor cells. Previous studies showed that phycocyanin from *Spirulina*-induced apoptosis reduced colony formation in the BC cell line [56]. There was marked necrosis in the tumor section from γT3-fed mice, which suggested that γT3 may induce tumor cell death.

In the current study, the metastasis of tumor cells from the primary site to liver tissues was observed even in mice fed with γT3, *Spirulina*, or the combination treatment. The findings suggest that supplementation with γT3, *Spirulina*, or combined treatment could not inhibit metastasis in this experimental model. However, there were no metastases to the lung, heart, and kidneys, which might be due to the limitation of the tumor cells’ invasive capacity from the primary tumor to these organs compared to the liver. In previous studies using the same mouse model of BC, it was reported that VEGF expression was significantly reduced in mice fed with TRF compared to the control group [57]. This shows that palm tocotrienols exhibit anti-angiogenic properties that may assist in tumor regression. However, in the current study, γT3 did not appear to inhibit metastasis to the liver.

Combination treatment using γT3 and *Spirulina* was found to be ineffective in producing anticancer or immunomodulatory effects. In fact, the response seems to negate the positive effects when each nutraceutical was used on its own. Although combined treatment of γT3 with *Spirulina* was not reported on previously, combination of T3 with other bioactive compounds were tested in several tumor models. For instance, combination of T3 and gemcitabine was reported to down-regulate NF-Kβ activity along with NF-Kβ regulated gene products, such as cyclin D1, c-Myc, VEGF, MMP-9, and CXCR4 in pancreatic cancer [58]. This combination also potentiated the anti-tumor activity of gemcitabine by inhibiting NF-Kβ and NF-Kβ regulated gene products, leading to the inhibition of proliferation, angiogenesis, and invasion [58]. However, it should be noted that the concentration of T3 used in the pancreatic cancer model was higher than what was used in the present study. This may account for the differences observed. *Spirulina* used in combination with other natural compounds was reported to exert anti-invasive effects against BC. For instance, combination of phycocyanin from *Spirulina* with indol-3-carbinol, resveratrol, isoflavone, curcumin, and quercetin was shown to downregulate CD44, and reduce migration (wound healing assay) and invasion (matrigel assay) of human BC cell lines [59].

BIRC5/Survivin is known as an apoptosis inhibitor [60], while Serbp1/Serpine-1/PAI-1 is associated with tumor invasion [61], and MIG6 [62] and Cadherin 13 [63] are known as tumor suppressors. Further studies using more genes associated with specific pathways would help in understanding the anticancer effects of combined γT3 and *Spirulina* against BC. BIRC5/Survivin is an inhibitor of apoptosis protein (IAP) and is overexpressed in a wide spectrum of tumors, including breast cancer [64,65]. Its main function includes inhibiting apoptosis and regulating mitosis, which is associated with carcinogenesis [60]. Apoptosis, or programmed cell death, involves caspases responsible for proteolytic cleavages that lead to cell death. Two mechanisms are involved in apoptosis, namely death receptor (a subgroup of tumor necrosis factor (TNF) superfamily activation) and mitochondrial stress apoptotic signaling pathways [61]. This indicates that BIRC5/Survivin modification could be therapeutic against cancer. In the current study, there were no significant (*p* > 0.05) changes in BIRC5 gene expression upon treatment with *Spirulina,* γT3, or combined treatment with *Spirulina* and γT3.

Another gene analyzed in this study was Serbp1/Serpine-1/PAI-1, a multifaceted proteolytic factor that functions as an inhibitor of the serine protease and plays an important role in signal transduction, cell adhesion, and migration [66]. High levels of Serbp1/Serpine-1/PAI-1 have also been consistently reported to predict poor prognosis in several types of human cancers [67,68] and are associated with tumor aggressiveness and poor patient outcomes [69]. In the current study, there were no significant (*p* > 0.05) changes in Serbp1/Serpine-1/PAI-1 gene expression upon treatment with *Spirulina,* γT3, and combined treatment with *Spirulina* and γT3.

MIG6 is a negative feedback regulator of receptors for tyrosine kinases, and the expression of this gene was down-regulated in human breast carcinomas, correlating with reduced overall survival of breast cancer patients [70,71]. Previous studies showed that MIG6 expression is reduced in skin, breast, pancreatic, and ovarian carcinomas [72]. In this study, MIG6 gene expression was significantly (*p* < 0.05) up-regulated upon treatment with γT3 as compared to the control. Previous studies showed that α-T_3_, γ-T3, and δ-T_3_ can up-regulate the expression of the MIG6 gene in a breast cancer cell line (MCF-7) [73]. Although the present study was based on the in vivo model, γT3 was still able to enhance tumor suppressor gene expression. In *Spirulina* and combined treatment, there were no significant (*p* > 0.05) differences in this gene expression.

Cadherin 13, also referred to as T- or H-cadherin, is expressed in multiple cell types in the breast gland, including myoepithelial, epithelial, and endothelial cells [74]. Characterization of Cadherin 13 suggests that it is not necessarily involved in adhesion, but instead is an adiponectin receptor [75] and adiponectin is secreted by adipocytes, which can sequester growth factors. Cadherin 13 is frequently silenced in different cancers, and it has long been considered a tumor suppressor [63]. In this study, γT3 significantly (*p* < 0.05) up-regulated the expression of Cadherin 13 compared to the control, which indicates that it enhanced the tumor suppressing effects and, thus, could inhibit tumor development. In the current study, there were no significant (*p* > 0.05) changes in Cadherin 13 gene expression upon treatment with *Spirulina* and combined treatment with *Spirulina* and γT3. On the whole, upon the combination of γT3 and *Spirulina,* there were no significant (*p* > 0.05) differences in the expression of the genes analyzed in this study compared to the control group.

## 5. Conclusions

The primary aim of the current research was to assess the immunomodulatory, anti-metastatic, gene expression analysis, and anticancer effects of combined γT3 and *Spirulina* treatment against BC using a syngeneic mouse model of BC. Combination of γT3 and *Spirulina* did not cause any significant differences in body weight, and there was no consistent suppression of tumor volume observed. Moreover, there were no significant differences in the T-cell population upon combined treatment with γT3 and *Spirulina* as compared to the control group. In contrast, γT3 or *Spirulina* treatment on its own caused a significant increase in the Th population and a significant decrease in Treg populations compared to the control. Metastasis to the liver was present in sections from the control and all treatment groups, suggesting that these nutraceuticals could not inhibit metastasis to the liver. There were more necrotic cells in sections from γT3-supplemented mice compared to combined treatment (γT3 + *Spirulina*), which suggested that the necrotic effect upon combination is most likely from the γT3. In conclusion, the combination of γT3 + *Spirulina* did not appear to have any synergistic anti-cancer or immunomodulatory effects in this mouse model of BC.

## Figures and Tables

**Table 1:** Experimental groups.

Treatment Groups	Supplementation (50 µL/Day)	Number of Animals	Tumor Induction
Control	Vehicle (soy oil)	6	Yes
***Spirulina*** alone	*Spirulina* (50 mg/kg body weight)	6	Yes
γT3 alone	γT3 (1 mg/day)	6	Yes
***Spirulina*** + γT3	*Spirulina* (50 mg/kg body weight) + γT3 (1 mg/day)	6	Yes
γT3: gamma-tocotrienol			

## Supplementary Materials

The following are available online at https://www.mdpi.com/article/10.3390/nu13072320/s1, Figure S1: The melting curve analysis of the target and reference genes, Figure S2: Dot plot distribution.
