# Supplementary material for: Evaluating Anticancer and Immunomodulatory Effects of Spirulina (Arthrospira) platensis and Gamma-Tocotrienol Supplementation in a Syngeneic Mouse Model of Breast Cancer
Source: Nutrients. 2021 Jul 6;13(7):2320. doi: 10.3390/nu13072320 (PMC8308567; doi:10.3390/nu13072320)
Supplement: Supplementary file 1 [file nutrients-13-02320-s001.zip › nutrients-1236640-supplementary.pdf]

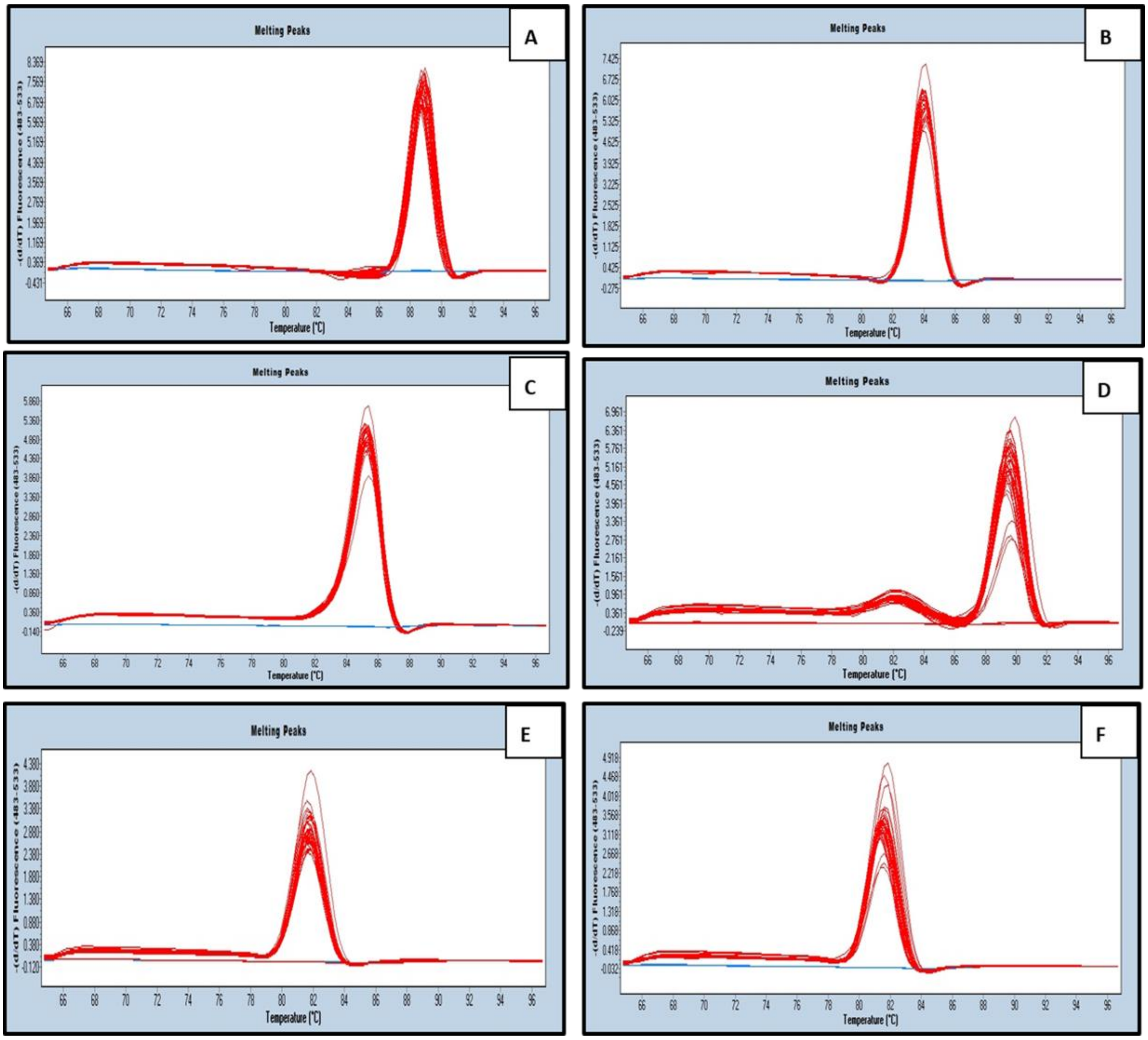

Figure S1. The melting curve analysis of the target and reference genes.

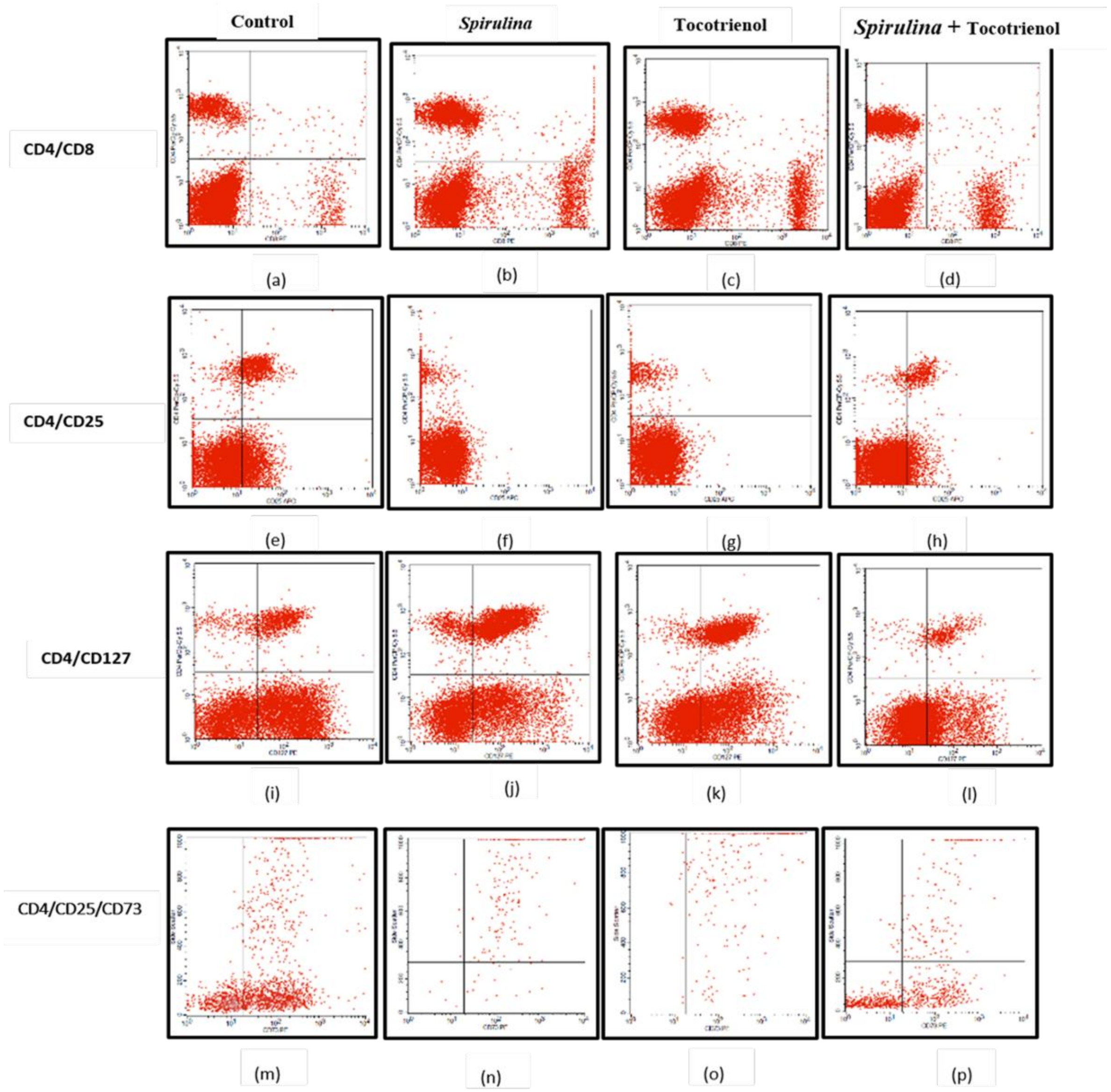

Figure S2. Dot plot distribution.
